# Intervention in Pregnangy Complicated with Albuminuria

**Published:** 1896-01

**Authors:** F. Paschal

**Affiliations:** San Antonio, Texas


For the Texas Medical Journal.

### INTERVENTION IN PREGNANCY COMPLICATED WITH ALBUMINURIA.

BY F. PASCHAL, M. D., SAN ANTONIO, TEXAS.

Read at the annual meeting of Southwest Texas Medical Society.

THIS paper is not intended as an exhaustive treatise on this subject; it is only for the purpose of eliciting an expression of opinion from the members present. We all know that there are few complications more dangerous to a pregnant woman than puerperal eclampsia. The etiology of this trouble is no better understood by us than it was by physicians twenty-five or fifty years ago.

The lesions found in cases dying from puerperal convulsions are epithelial, and interstitial nephritis; interstitial cirrhosis of the liver with infarcts; considerable development of the connective tissue in the uterus or tubes; inflammation of the cardiac parenchyma; and considerable distension of the cerebral capillaries. This shows that all of the organs are affected in eclampsia, and that the appearances of an acute intoxication are present. That the cause of this intoxication is due to uneliminated effete matters, principal amongst which is urea—is probable. Therefore, the treatment should be preventive, rather than than empirical. As it is the rule for albumen to be present in women attacked by puerperal convulsions, it should lead us to examine the urine of every pregnant woman whom we are engaged to attend, and this examination should be repeated frequently; and if albumen is present, strict diet should be enforced and other measures employed to eliminate effete matters. If, however, in spite of treatment the patient's condition does not improve, intervention should be employed and labor induced any time after the seventh month, or as the emergency of the case may demand, for it is dangerous to allow a woman to go to full term when the urine continues with albumen, or who has other symptoms of uremia.

The danger attending the induction of premature labor is practically nothing, and while a premature child is harder to raise, this should not interfere with relieving the mother of the dangers that threaten her. Tanner gives the statistics of forty-four cases of induced premature labor, extending over a period of ten years.

The mortality of the mothers was 2.2 per cent., and of the children, 18 per cent. A single death was independent of the method employed, the mother dying of pernicious anæmia, thus making the mortality really nothing. At Leopold's clinic, during three years and a half, there were eighty-one induced premature labors. The mortality for the mothers was nothing. The labors were induced for contracted pelves, but the mortality should not be greater if it were done for albuminous complications. In order to impress upon us the importance of this subject, I will cite the two following cases—typical of others—occurring in my practice:

I was engaged to attend a woman two months before her confinement, and found the urine with a considerable quantity of albumen. She had headache, blurred vision, puffed eyelids, nausea and vomiting, swollen feet and legs. Diet and treatment did not improve her condition. Labor was induced about the seven and one-half months without any bad consequences to mother or child.

In the second case, I was called to see a primipara who had engaged a physician three months before her confinement. He had not examined the urine. I found her in convulsions, having had her first one at 5 a. m.; I was called at 1:30 p. m. She had been given morphine and the usual chloroform administered. I delivered her, by version, of a live child, but the patient never came out of profound coma, and she died a few hours after delivery.

Is it not reasonable to suppose that had the proper urinary examination been made in this case, and premature labor induced, that the mother might have "felt for her first born, and a house turned into rejoicing instead of mourning?"

It seems to me that in such cases there should be no unnecessary delay, or trusting to luck, thereby endangering the life, or permanently injuring the woman's health from diseased kidneys, but that we should all appreciate the responsibilities that rest upon us in engaging to attend labor cases, and look carefully to the safety of two lives entrusted to our care.

